# Characterization of an Archaeal Two-Component System That Regulates Methanogenesis in *Methanosaeta harundinacea*

**DOI:** 10.1371/journal.pone.0095502

**Published:** 2014-04-18

**Authors:** Jie Li, Xin Zheng, Xiaopeng Guo, Lei Qi, Xiuzhu Dong

**Affiliations:** 1 State Key Laboratory of Microbial Resources, Institute of Microbiology, Chinese Academy of Sciences, Beijing, China; 2 University of Chinese Academy of Sciences, Beijing, China; Universidad de Costa Rica, Costa Rica

## Abstract

Two-component signal transduction systems (TCSs) are a major mechanism used by bacteria in response to environmental changes. Although many sequenced archaeal genomes encode TCSs, they remain poorly understood. Previously, we reported that a methanogenic archaeon, *Methanosaeta harundinacea*, encodes FilI, which synthesizes carboxyl-acyl homoserine lactones, to regulate transitions of cellular morphology and carbon metabolic fluxes. Here, we report that *filI*, the cotranscribed *filR2*, and the adjacent *filR1* constitute an archaeal TCS. FilI possesses a cytoplasmic kinase domain (histidine kinase A and histidine kinase-like ATPase) and its cognate response regulator. FilR1 carries a receiver (REC) domain coupled with an ArsR-related domain with potential DNA-binding ability, while FilR2 carries only a REC domain. In a phosphorelay assay, FilI was autophosphorylated and specifically transferred the phosphoryl group to FilR1 and FilR2, confirming that the three formed a cognate TCS. Through chromatin immunoprecipitation–quantitative polymerase chain reaction (ChIP-qPCR) using an anti-FilR1 antibody, FilR1 was shown to form *in vivo* associations with its own promoter and the promoter of the *filI-filR2* operon, demonstrating a regulatory pattern common among TCSs. ChIP-qPCR also detected FilR1 associations with key genes involved in acetoclastic methanogenesis, *acs4* and *acs1*. Electrophoretic mobility shift assays confirmed the *in vitro* tight binding of FilR1 to its own promoter and those of *filI-filR2*, *acs4*, and *mtrABC*. This also proves the DNA-binding ability of the ArsR-related domain, which is found primarily in Archaea. The archaeal promoters of *acs4*, *filI*, *acs1*, and *mtrABC* also initiated FilR1-modulated expression in an *Escherichia coli lux* reporter system, suggesting that FilR1 can up-regulate both archaeal and bacterial transcription. In conclusion, this work identifies an archaeal FilI/FilRs TCS that regulates the methanogenesis of *M. harundinacea*.

## Introduction

Methanogenesis is a major contributor to global warming, and methanogens are the only organisms known to perform this metabolism [1]. Acetate-derived methane contributes about 70% of the global methane production and is produced by acetoclastic methanogens such as *Methanosarcina* and *Methanosaeta*. Although these archaea possess prokaryotic cells, their genetic machinery for replication, transcription, and translation more closely resembles that of Eukarya than Bacteria [2]. Methanogens are archaea that are distributed in diverse anoxic environments and can develop complex regulation mechanisms in response to environmental changes [3], [4].

Two-component signal transduction systems (TCSs) are one of the principal means by which bacteria respond to environmental changes [5]–[7]. Gao and Stock summarized the diverse bacterial TCSs, indicating that the typical TCS comprises a membrane-bound sensor histidine kinase (HK) and a cognate response regulator (RR) and catalyzes a phosphotransfer between the two [8]. Typically, upon sensing environmental stimuli, the HK is autophosphorylated at a conserved histidine residue. The phosphoryl group is then transferred to its cognate RR at a conserved aspartate residue. In prototypical HKs, the cytoplasmic kinase core consists of a well-conserved C-terminal HK-like ATPase (HATPase) and a less-conserved histidine kinase A (HisKA) domain [9], which can be coupled to an overwhelmingly diverse array of sensory domains, including PAS, GAF, and HAMP domains, enabling HKs to sense a wide variety of stimuli. Characteristic RR superfamily proteins contain a receiver (REC) domain, which is linked to variable effector domains that mediate nucleic acid binding or enzymatic and protein/ligand-binding activity [8]. Moreover, approximately 17% of RRs exist as stand-alone REC domains that lack an effector domain [8], [10], [11]; these RRs usually implement a more intricate regulation of a TCS [12], [13].

TCS genes are found in many sequenced archaeal genomes; however, they are poorly studied [7], [14], [15]. *Methanosaeta harundinacea* 6Ac is an obligate acetoclastic methanogenic archaeon that was isolated from an upflow anaerobic sludge blanket reactor [16]. It contributes to granule formation and the efficacy of waste removal in the reactor. Previously, we determined that FilI, a bacterial LuxI homolog from *M. harundinacea*, synthesized the quorum sensing (QS) signal molecules carboxyl-acyl homoserine lactones (AHLs), which regulate the cell morphological transition from short cells to filaments and carbon metabolic flux from biomass formation to methane production [17].

In this work, based on *in silico* analysis, *filI*, the cotranscribed gene *filR2*, and the adjacent gene that we tentatively named *filR1* were predicted to encode the only TCS in this archaeon. Then, through a combination of *in vivo* and *in vitro* approaches, FilI and the two FilRs were shown to possess many of the properties of bacterial TCSs. Moreover, they were found to be involved in the regulation of methanogenesis in *M. harundinacea*.

## Results

### *In silico* analysis predicted a putative TCS in *M. harundinacea*

**Figure 1:**
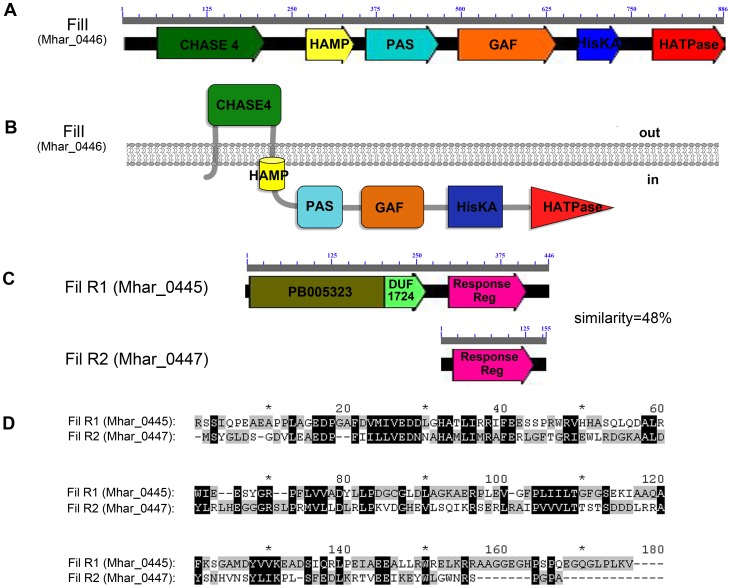
Schematic representation of the domain structures of FilI and FilR proteins. (A) Domain structure of FilI analyzed using programs of Pfam and NCBI blast. (B) Location analysis of domains in FilI through the programs of TMHMM, TMpred and SOSUI. (C) Domain structures of FilR1 and FilR2 were analyzed by programs of Pfam and NCBI blast. In addition, the amino acid identity (%) for the aligned fragments of FilR1 and FilR2 is shown on the right. (D) Protein sequence alignment of the REC domains of FilR1 (C-terminal 276–446aa) and FilR2 (the whole length) by software GeneDoc. Identical amino acids are shown with a black background, while similar amino acids are shown with a gray background.

To gain a general view of the TCSs in *M. harundinacea* 6Ac, bacterial prototypical kinase core domains of HKs and REC core domains of RRs were used as probes to query the genome (CP003117) [17]. The search found only three putative HKs (Mhar_0446, Mhar_0936, and Mhar_1766) with characteristic cytoplasmic HisKA and HATPase domains, and it identified five RRs (Mhar_0169, Mhar_0445, Mhar_0447, Mhar_1520, and Mhar_2042) with REC core domains. These results were consistent with those of the P2CS and MiST2 databases [18], [19]. Further, transmembrane regions of the three putative HKs were analyzed using three programs: TMHMM (http://www.cbs.dtu.dk/services/TMHMM/) [20], TMpred (http://www.ch.embnet.org/software/TMPRED_form.html), and SOSUI (http://bp. nuap.nagoya-u.ac.jp/sosui/sosui_submit.html) [21]. All three programs revealed that only Mhar_0446 was likely to possess the two transmembrane regions that are common in prototypical membrane-bound HKs (Figure 1A, 1B). Next, functional domains of the five RRs were analyzed using the Pfam database (http://pfam.sanger.ac.uk/) [9]. Mhar_0447 and Mhar_1520 were stand-alone RRs that possessed only the REC domain (Figure 1C), while Mhar_0169 and Mhar_2042 had REC domains that were fused with PAS sensory domains, which is an unusual RR organization in which the REC domain is fused to a ligand-binding domain [10]. Only Mhar_0445 appeared to encode a typical TCS RR with a REC core domain coupled with PB005323, a predicted ArsR-related domain with potential DNA-binding ability (Figure 1C). Moreover, Mhar_0446 and Mhar_0447 could constitute an operon because they are only separated by a short intergenic distance of 37 bp and are adjacent to Mhar_0445. Therefore, the three proteins might constitute the only typical TCS in *M. harundinacea*.

Mhar_0446 encodes a protein of 886 amino acid residues that exhibits 39.6% identity to AhlI, a LuxI family autoinducer synthase of *Erwinia chrysanthemi* [17]. The recombinant His_6_-tagged protein had carboxyl-AHL synthase activity; therefore, Mhar_0446 was named FilI [17]. Using the Pfam database, six functional domains were predicted in FilI, including a CHASE4 domain, a HAMP (potential signal transmission) domain, a PAS intracellular stimuli sensory domain, a GAF intracellular stimuli sensory domain, a C-terminal HisKA domain, and an HATPase domain (Figure 1A, 1B). Moreover, the transmembrane region prediction programs TMHMM, TMpred, and SOSUI indicated that the CHASE4 domain and the HAMP domain are extracytoplasmic or transmembrane, while the other four domains are all cytoplasmic (Figure 1B). In addition, the AHL synthase activity of FilI suggested a potential connection between the predicted FilI/FilRs TCS and quorum sensing (QS) in *M. harundinacea*.

An alignment of the two RRs in this putative TCS (Figure 1C), named FilR1 and FilR2, indicated a sequence similarity of 48% between the C-terminal REC domains of FilR1 and FilR2 (Figure 1D). Moreover, according to the domain compositions, FilR1, with a potential DNA-binding domain, was predicted to be the direct effector of this putative TCS. FilR2, as a stand-alone RR, was predicted to be involved in yet-unknown regulation mechanisms.

### *filI* and *filR2* constituted an operon and were cotranscribed

**Figure 2:**
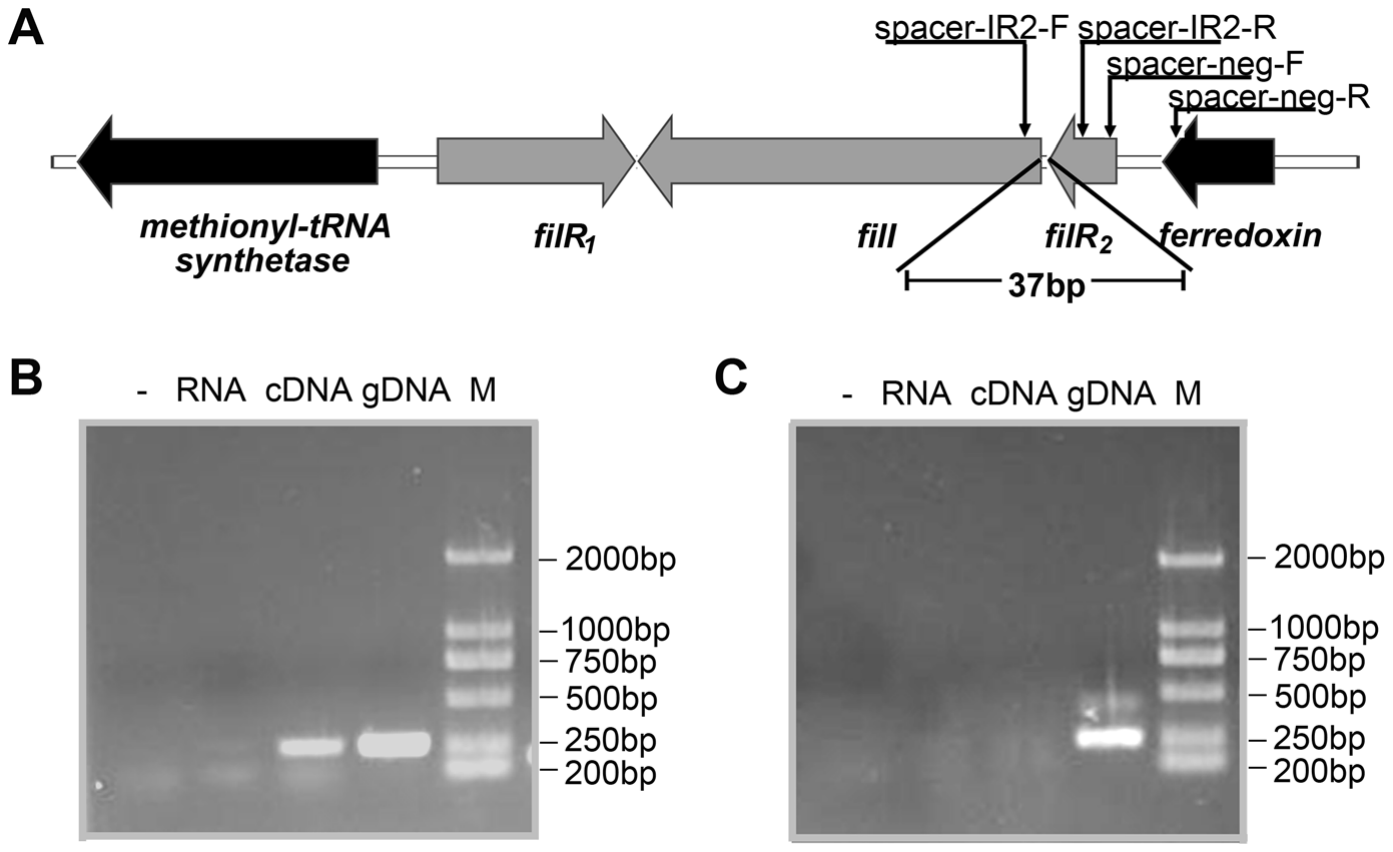
Cotranscription of *filI* and *filR2* in *M. harundinacea*. (A) Schematic arrangement of *filI* and *fliR2* in the genome. (B) Agarose gel electrophoresis of PCR products amplified from the intergenic region between *filI* and *filR2*. (C) The intergenic spacer between *filR2* and its upstream gene encoding a ferredoxin using the respective template labeled at the top of each gel: -, no DNA; RNA, total RNA extracted form *M. harundinacea* cells; cDNA, reverse transcripts from the total RNA; gDNA, genomic DNA of *M. harundinacea*. M, DL2000 marker with the sizes shown at the right. Primers spacer-IR2-R/F and spacer-gen-F/R were used for PCR reactions in (B) and (C), respectively.

The *in silico* analysis predicted that *filR2* and *filI* constitute an operon from which *filR1* is divergently transcribed (Figure 2A). To test this prediction, a reverse transcription-polymerase chain reaction (RT-PCR) assay was performed using the total RNA of *M. harundinacea* as a template. As shown in Figure 2B, a PCR product was amplified from the cDNA of the *filI*-*filR2* intergenic region, indicating the cotranscription of *filI* and *filR2* (Figure 2B). As a control, no amplification was produced from the intergenic region between *filR2* and the downstream ferredoxin gene (Figure 2C).

### Autophosphorylation of FilI and phosphotransfer among FilI and the FilRs

**Figure 3:**
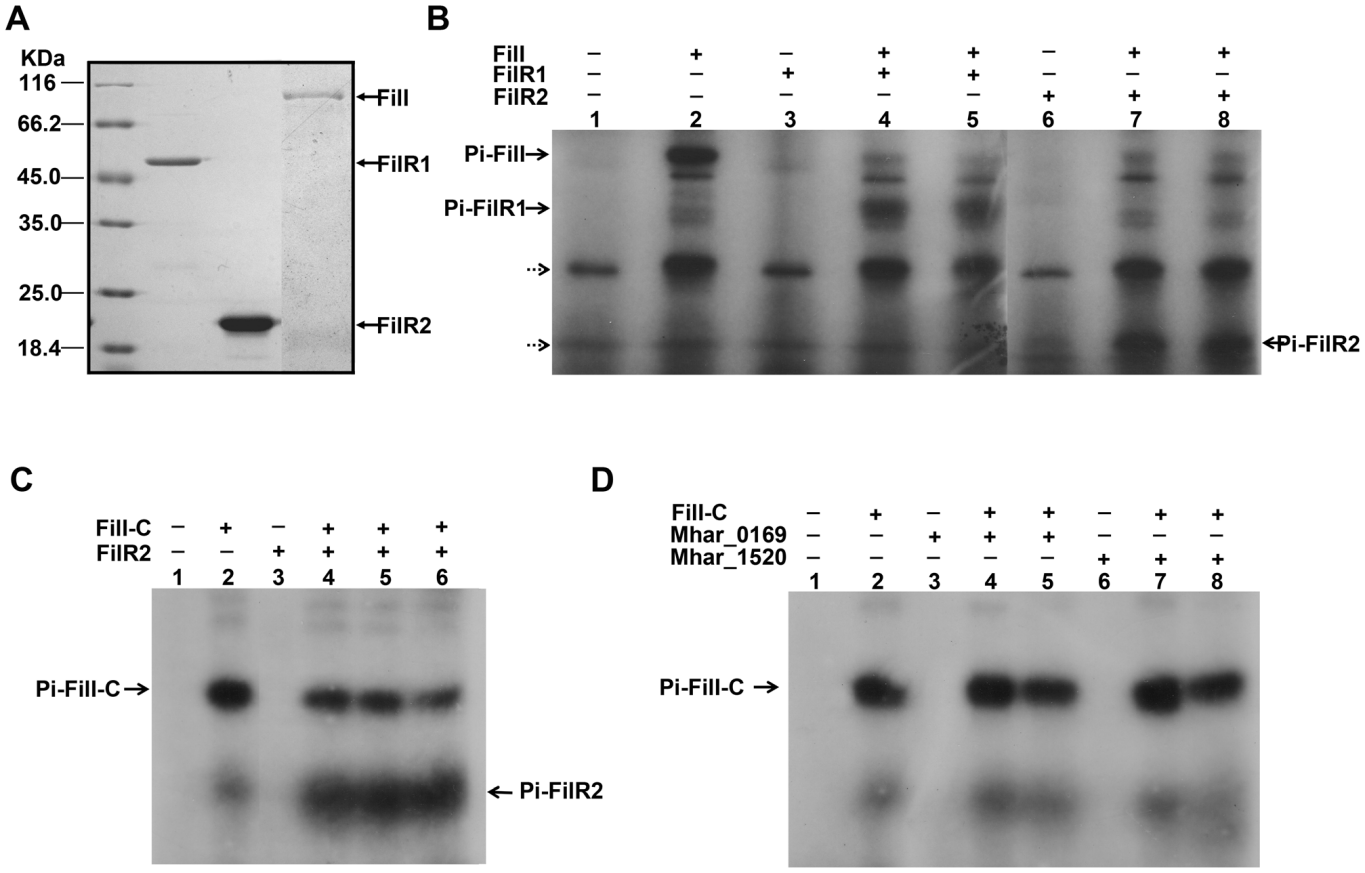
Assays of the autophosphorylation of FilI and phosphotransfer from FilI to FilR1 and FilR2. (A) SDS-PAGE of the recombinant his-tagged FilI, FilR1 and FilR2 purified from *E. coli*. (B) An autoradiogram visualized the autophosphorylation of the recombinant entire FilI protein (2 µg) after incubation for 45 min at 37°C in the presence of [γ-^32^P]ATP (lane 2) and without FilI as the negative control (lane 1). Phosphotransfer was assayed by addition of 4 µg of His-tagged RRs into the autophosphorylation reaction of FilI for certain times before being visualized on SDS-PAGE. Lane 3 and 6, negative controls of the phosphotransfer reactions without FilI included. Lane 4 and 5, FilR1 phosphorylated for 2 and 5 min, respectively; lane 7 and 8, FilR2 phosphorylated for 2 and 5 min, respectively. Dotted arrows indicate nonspecific bands generated from [γ-^32^P]ATP. (C) An autoradiogram visualized the autophosphorylation of the N-terminal truncated FilI (FilI-C) and phosphotransfer assay under the same treatments as the entire protein. Lane 1, no truncated FilI included; lane 2, FilI-C incubated with [γ-^32^P]ATP for 45 min; lane 3, FilR2 incubated with [γ-^32^P]ATP for 10 min; lane 4, 5 and 6, FilR2 phosphorylated for 2, 5 and 10 min, respectively. (D) An autoradiogram visualized the autophosphorylation of FilI-C and phosphotransfer assay to other regulators under the same treatments as the entire protein. Lane 1, no truncated FilI included, lane 2, FilI-C incubated with [γ-^32^P]ATP for 45 min; lane 3 and 6, proteins Mhar_0169 and Mhar_1520 incubated with [γ-^32^P]ATP for 10 min, respectively; lane 4 and 5, Mhar_0169 phosphorylated for 10 and 30 min, respectively; lane 7 and 8, Mhar_1520 phosphorylated for 10 and 30 min, respectively. Solid arrows indicate the phosphorylated proteins: Pi-FilI, Pi-FilI-C, Pi-FilR1 and Pi-FilR2.

A functional TCS relies on the autophosphorylation of the HK and a phosphotransfer from the HK to the cognate RR [8]. To determine if these activities are present in the predicted FilI/FilRs TCS system, recombinant FilI, FilR1, and FilR2 were purified from *Escherichia coli* (Figure 3A). FilI was readily autophosphorylated within 45 min of incubation with [γ-^32^P] ATP (Figure 3B). Phosphorylated FilI was capable of robust phosphoryl group transfers to both FilR1 and FilR2 within a short time (Figure 3B). Autophosphorylation of the truncated FilI protein, containing only the C-terminal kinase core domain, was also detected. This domain was able to phosphorylate FilR2, but its efficiency was lower than that of the entire FilI protein (Figure 3C). In contrast, the truncated FilI only weakly phosphorylated the other two potential RR transcriptional regulators Mhar_0169 and Mhar_1520 after 30 min (Figure 3D). These results demonstrated the specificity of the phosphorelay from FilI to FilR2 and to FilR1, and demonstrated the activity of the FilI HK domain. Further, the FilI-synthesized carboxyl-AHLs (*N*-carboxyl-C10-HSL, m/z 318, or *N*-carboxyl-C12-HSL, m/z 346) were added directly to the autophosphorylation reaction of FilI and phosphortransfer reactions to FilRs. However, very weak enhancing effects were observed (Figure S1). Therefore, the FilI-synthesized carboxyl-AHLs did not have an evident direct effect on the autophosphorylation of FilI or phosphotransfer to FilRs in the *in vitro* experiment.

### Chromatin immunoprecipitation-quantitative PCR (ChIP-qPCR) assay of the *in vivo* associations of FilR1 with the promoters of the TCS system and the key genes of methanogenesis

**Figure 4:**
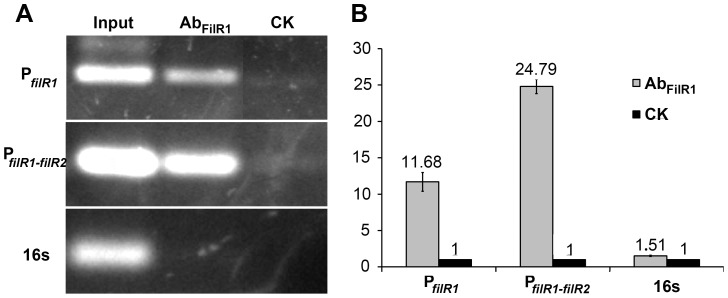
ChIP assays showed FilR1 binding to the promoters of its own (P*_filR1_*) and *filI-filR2* operon (P*_filR1-filR2_*) inside the cells of *M. hurandiacea* 6AC. (A) PCR products of the promoters of its own (P*_filR1_*) and *filI-filR2* operon (P*_filR1-filR2_*) were amplified from the anti-FilR1 antibody immunoprecipitated DNA (Ab_FilR1_), and input DNA sample before immunoprecipitation (Material and Methods) as a positive control (Input). Almost no PCR products were amplified from the mock-IP DNA (CK) samples. (B) qPCR detected the enrichment folds of the DNA fragments in anti-FilR1 antibody immunoprecipitated DNA (Ab_FilR1_, gray bar) over mock-IP control (CK, black bar). PCR amplifications were performed using the specific primers for the promoter regions of *filR1* (P*_filR1_*) and *filI-filR2* operon (P*_filI-R2_*). An intragenic DNA fragment of the16S rRNA gene (16 s) was included as the negative control.

Because of the lack of a genetic system for *M. hurandinacea* 6Ac, the *in vivo* TCS action of FilI and FilR1 was determined using ChIP assays. *M. harundinacea* 6Ac possesses a thick cell envelope; therefore, a novel method to gently lyse the cells with dithiothreitol (DTT) treatment at alkaline pH was developed from a procedure for *Methanospirillum* [22]. As shown in Figure 4A, the promoter regions of *filR1* and the *filI-filR2* operon were enriched in the anti-FilR1 antibody–immunoprecipitated DNA (lane AbFilR1) but not in the mock-IP control DNA (lane CK). Moreover, compared with the mock-IP DNA (CK), qPCR indicated 11.7- and 24.8-fold enrichments for the promoters of *filR1* and the *filI-filR2* operon in the anti-FilR1 antibody–immunoprecipitated DNA (Figure 4B), respectively. In the same experiment, there was no enrichment for a fragment of similar length from the 16S rRNA gene (Figure 4). Therefore, ChIP-PCR and ChIP-qPCR assays verified the *in vivo* association of FilR1 with its promoter and the promoter of the *filI-filR2* operon, a common characteristic of TCSs.

**Figure 5:**
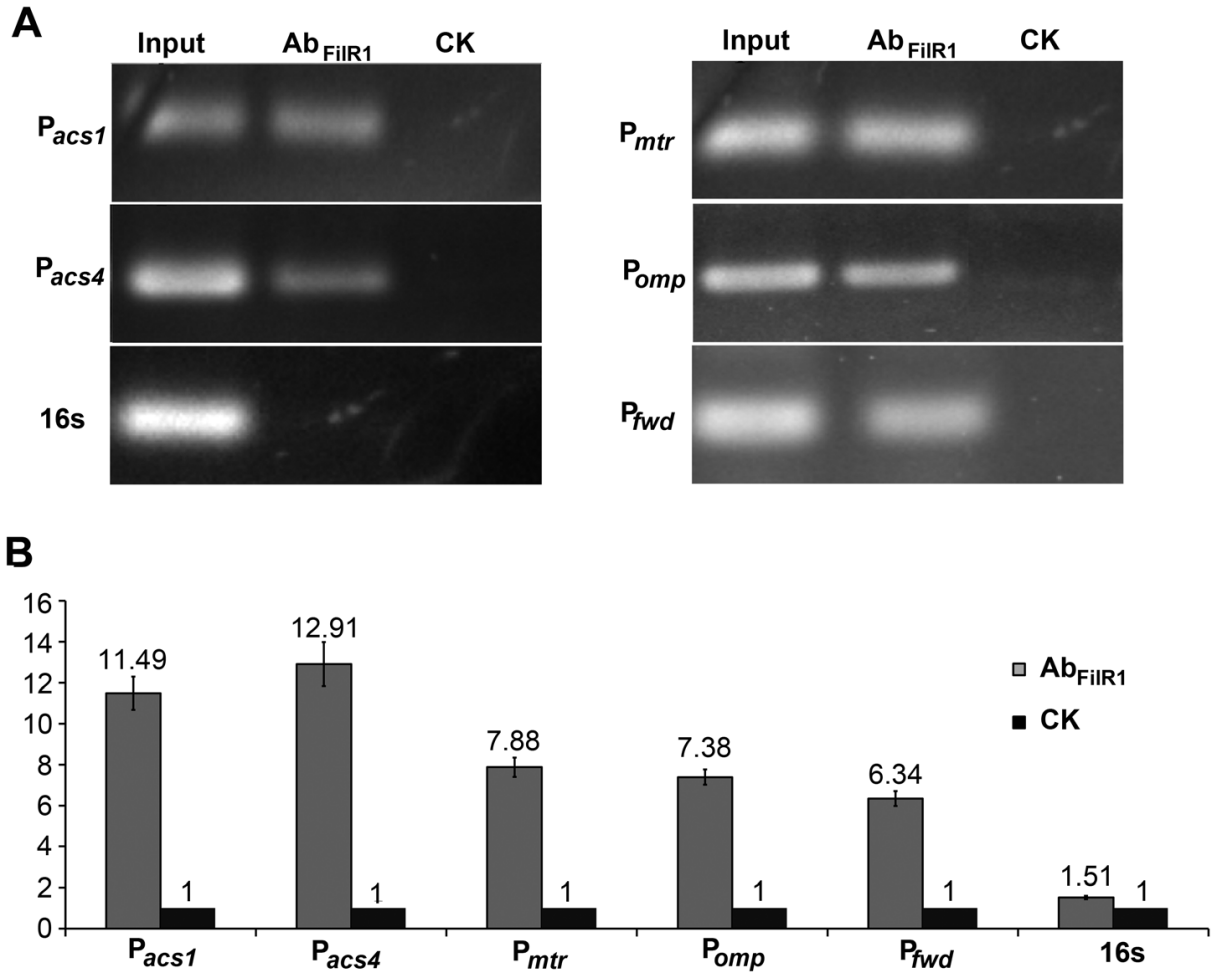
ChIP-PCR and ChIP-qPCR detected FilR1 associations with the promoters of genes for methanogenesis inside *M. harundinacea* cells. (A) PCR products amplified with the indicated primers using anti-FilR1 antibody immunoprecipitated DNA (AbFilR1) as a template and mock-IP DNA as a negative control (CK). (B) qPCR assays detected the enrichment of DNA fragments in anti-FilR1 antibody immunoprecipitated samples (AbFilR1, gray bar) over mock-IP negative control samples (CK, black bar). P*_acs1_*, promoter region of operon *acs1*; P*_acs4_*, promoter region of *acs4*; P*_mtr_*, promoter region of operon *mtr*; P*_fwd_*, promoter region of the operon *fwdCABD*; P*_omp_*, promoter region of *omp* and 16s, intragenic DNA fragment of 16S rDNA used as the control.

Previously, FilI-synthesized AHLs were found to regulate a transition of the cellular morphology from short to long cells and a carbon metabolic flux leading to the formation of more methane and less cellular biomass from acetate in *M. harundinacea* [17]. To test whether the FilI cognate RR FilR1 was involved in the regulation of methanogenesis, associations of FilR1 with the key genes in acetoclastic methanogenesis were assayed using the same protocol. As shown in Figure 5, the promoter regions of the *acs1* operon, *acs4* gene, *mtr* operon, *fwdCABD* operon, and *omp* gene (Table S1) were all amplified by PCR assays from the anti-FilR1 antibody–immunoprecipitated DNA samples (Figure 5A). Also, qPCR indicated 11- and 13-fold enrichments of the promoter regions of the *acs1* operon and the *acs4* gene, respectively, and modest enrichment of the *mtr* and *fwd* operons and the *omp* gene (6.34- to 7.88-fold enrichment) (Figure 5B). Therefore, FilR1 interacted with the promoters of a number of genes that are essential for methanogenesis in *M. harundinacea* 6Ac as summarized in Table S1.

### FilR1 bound *in vitro* to the promoters of the TCS system and key genes of methanogenesis

**Figure 6:**
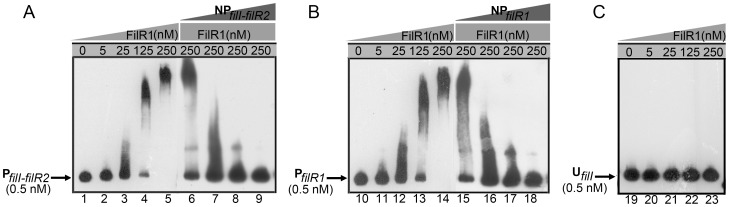
EMSAs showed FilR1 binding to the promoters of its own and the operon *filI-filR2*. Purified recombinant FilR1 protein was incubated with 0.5-labeled DNA in the standard binding reaction mixture at 25°C for 20 min, and then run on a native PAGE. Concentration of purified FilR1 protein was shown at the top of each lane. Unlabeled *filI-filR2* promoter (NP*_filI-filR2_*) was used as a competitor substrate of FilR1, which was added at the final concentrations of 5, 25, 125 and 250 nM in lane 6 and 15, 7 and 16, 8 and 17, and 9 and 18, respectively. (A) P*_filI-R2_*, promoter of the *filI-filR2* operon; (B) P*_filR1_*, promoter of *filR1*, and (C) U*_filI_*, an internal DNA fragment of gene *filI*.

Next, electrophoretic mobility shift assays (EMSAs) were performed to confirm the *in vitro* binding of FilR1 to the gene promoters studied above. Using a biotin-labeled DNA amplified from the tested gene promoters as probes, DNA-protein complexes with the recombinant FilR1 were detected by native polyacrylamide gel electrophoresis (PAGE). As shown in Figure 6, FilR1 bound efficiently to its promoter and that of the *filI-filR2* operon in a dose-dependent manner, demonstrating its auto-regulation and regulation of the *filI-filR2* operon.

**Figure 7:**
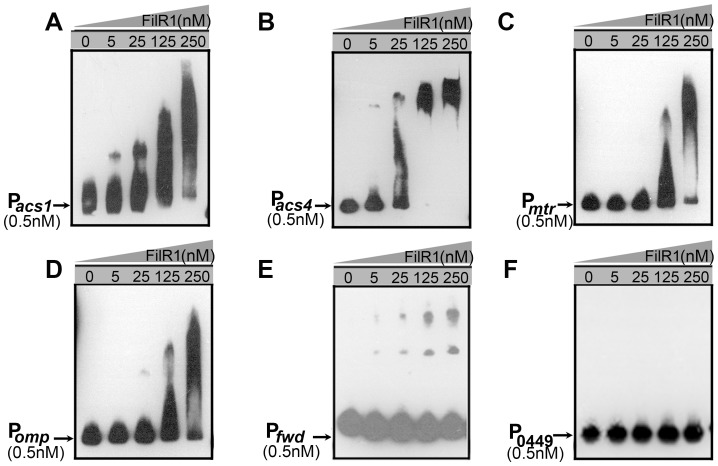
EMSAs showed FilR1 binding to the promoters of genes key to methanogenesis in *M. harundinacea*. Purified recombinant FilR1 protein was incubated with 0.5-labeled DNA in the standard binding reaction mixture for 20 min at 25°C and then run on a native PAGE. Concentration of purified FilR1 protein used was shown at the top of each lane. (A) P*_acs1_*, promoter of the *acs1* operon; (B) P*_acs4_*, promoter of *acs4*; (C) P*_mtr_*, promoter of the *mtr* operon; (D) P*_fwdCABD_*, promoter of the *fwdCABD* operon; (E), P*_omp_*, promoter of *omp*; and (F) P*_Mhar_0449_*, promoter of Mhar_0449.

Similarly, EMSA also detected the *in vitro* binding of FilR1 to promoters of the *acs1* operon, *acs4* gene, *mtr* operon, *fwdCABD* operon, and *omp* gene (Figure 7). However, no binding was detected for the promoter of Mhar_0449 and an internal fragment of *filI*, which served as negative controls (Figure 7 and data not shown). These data further confirmed the direct regulation acetoclastic methanogenesis and the methyl oxidative shunt by FilR1; these findings were consistent with the *in vivo* binding results determined by the ChIP assays described above. In addition, the phosphorylated FilR1 via phosphotransfer from FilI exhibited an obviously enhanced DNA-binding affinity to the promoters of the *acs1* and *fwdCABD* operons (Figure S2), implying the possible positive regulation of methanogenesis by the FilI/FilRs TCS in *Methanosaeta*.

However, EMSA did not detect the binding of FilR1 to the promoters of some other differentially expressed genes in the QS-mediated transition. These genes included the *cdhCD* and *cdhBEA* operons, which encode the key enzyme complex for the acetoclastic reaction; the *mcr* operon, which encodes the methyl-CoM reductase; and the *fpo* operon, which encodes the only membrane-associated protein complex for electron transfer in *Methanosaeta* (data not shown). Therefore, the expression of these genes is probably not directly regulated by FilR1.

The ArsR-related domain is found primarily in Archaea, and it has not yet been shown to bind DNA. This work also provides the first experimental evidence of the DNA-binding ability of the ArsR-related domain.

### An *ex vivo E. coli* promoter-reporter system detected FilR1 regulation of the genes involved in methanogenesis

An *E. coli*-based promoter-reporter *ex vivo* system [23], [24] was used to further examine the regulation of FilR1. First, the *filR1* open reading frame (ORF) with a prototypical *E. coli* ribosomal binding site sequence was cloned into the expression plasmid p184 (abbreviation of pACYC184) to generate the plasmid pFilR1 (abbreviation of pACYC-FilR1) for heterogeneous expression of FilR1 protein in *E. coli*. Western blots with anti-FilR1 antibody detected a protein band of 54 kDa, the predicted size of FilR1, in extracts of transformed *E. coli* (data not shown). Meanwhile, the examined promoters were fused upstream of a promoterless *lux* operon inserted into pCS26-Pac to construct a variety of methanogenic promoter-*lux* reporter plasmids denoted as pO^x^-lux (x: the tested genes) (Table 1). pFilR1 and each of the pO^x^-lux promoter-*lux* reporter plasmids were cotransformed into *E. coli*. p184 was cotransformed with the pO^x^-lux reporter plasmids as a negative control. As shown in Table 1, although the *mtr*, *fwd*, and *cdhCD* operons and the *acs4* gene possessed archaeal promoters, they were recognized by *E. coli* RNA polymerase and promoted the transcription of the downstream *lux* gene, yielding luminescence comparable to that of bacterial promoters. Coexpression with FilR1 caused a remarkable stimulation of the *acs4* (46-fold) and *filI-filR2* (33-fold) promoters. FilR1 also increased the bioluminescence from the *omp* (13-fold), *filR1* (8-fold), *acs1* (6-fold), and *mtr* (3.7-fold) promoters (Table 1), suggesting that FilR1 regulated these genes as well. In contrast, the FilR1-stimulated expression by the promoter of the *fwdCABD* operon was increased less than 2-fold in this promoter-reporter system (Table 1). The *fwdCABD* promoter also possessed low affinity to FilR1 in EMSA (Figure 7D). Similarly, FilR1 did not affect the expression from the promoters of *cdhCD*, *cdhBEA*, *mcrBD*, *mcrCA*, *fpo*, and *RNA pol*, which are genes whose expression was not directly regulated by FilR.

**Table 1:** Expression of the archaeal promoters in the *E. coli ex vivo* reporter system.

	Bioluminescence^a^			Fold stimulation by FilR1^c^		
Reporter plasmids^b^	−	+p184	+pFilR1	+p184/−	+pFilR1/−	+pFilR1/+p184
pCS26-Pac	204±22	179±33	150±6	0.9	0.7	0.8
pO*^filR1^*-lux	632±63	437±68	3334±517	0.7	5.3	7.6
pO*^filI-filR2^*-lux	2415±357	1985±466	66330±6361	0.8	27.5	33.4
pO*^Acs1^*-lux	1218±24	1169±11	7361±23	1.0	6.0	6.3
pO*^Acs4^*-lux	170631±8043	169013±4054	7758086±61087	1.0	45.5	45.9
pO*^Mtr^*-lux	911854±14512	879377±60268	3247045±67755	1.0	3.6	3.7
pO*^Omp^*-lux	357±78	332±58	4181±419	1.0	11.7	12.6
pO*^FwdCABD^*-lux	26060±4240	29650±1415	45848±1864	1.1	1.8	1.6
pO*^CdhCD^*-lux	45059±641	18814±30	32223±3715	0.4	0.7	1.7
pO*^CdhBEA^*-lux	195±44	201±4	144±2	1.0	0.7	0.8
pO*^McrBD^*-lux	6657±174	6151±64	6963±424	0.9	1.1	1.1
pO*^McrCA^*-lux	494±7	554±288	668±199	1.1	1.4	1.2
pO*^Fpo^*-lux	2592±519	2994±365	1567±94	1.5	0.6	0.5
pO*^RNA pol^*-lux	171±44	157±11	160±9	0.9	0.9	1.0

a. Values are shown as relative light units and the average of at least three independent readings.

b. Genes and operons shown in each reporter plasmid are listed in Table S1.

c. Fold difference of the luciferase activity are calculated from those determined for *E. coli* strain carrying plasmid pairs of FilR1-vacant p184 plus pO^x^-lux over that of the strain carrying pOx-lux alone (+p184/−), strain with FilR1 plus pO^x^-lux over that with pO^x^-lux alone (+pFilR1/−), and strain with FilR1 plus pO^x^-lux over that with FilR1-vacant p184 plus pO^x^-lux (+pFilR1/+p184).

To determine if archaeal promoters share the characteristics of bacterial promoters and, therefore, can be recognized by *E. coli* RNA polymerase, we first predicted the promoters in the above-studied genes using the Neural Network Promoter Prediction program (http://www.fruitfly.org/seq_tools/promoter.html) [25] (Table S2). In addition to a typical archaeal promoter, a bacteria-like promoter was found upstream of the *mtr* coding region (Figure S3). Coincidently, the highest bioluminescence was detected for the *mtr* and *acs4* promoters in the *E. coli* promoter-reporter system (Table 1). Furthermore, a GC substitution was made for a six base-AT tract in the predicted promoters of *mtr*, *acs4*, *filR1*, and *filI-filR*. The bioluminescence from the mutated *filI-filR2* and *mtr* promoters was reduced 6.4- and 31.5-fold, respectively, and FilR1-induced bioluminescence was also reduced (Table S3). The bioluminescence was not affected by mutations in the *acs4* and *filR1* promoters; this observation can probably be attributed to an incorrect promoter prediction. This indicates that *E. coli* RNA polymerase can recognize some archaeal promoters.

## Discussion

TCSs are distributed widely in bacteria and are well characterized [7], [14], [19]. They are abundant in free-living species like *E. coli* and *Bacillus subtilis*, which contain 30 and 36 TCSs [19], respectively, but they are absent in many parasites, such as *Mycoplasma genitalium* and *Mycoplasma pneumonia* [14], [15]. Because TCSs enable organisms to adapt to environments, an abundance of TCSs is considered a measure of the bacterial IQ [26]. Authentic genes of archaeal TCSs are only found in some Euryarchaeota and Thaumarchaeota genomes, and they are found in neither Crenarchaeota nor Nanoarchaeota genomes [14], [15]. In these phyla, one-component systems of a single protein containing both sensor and regulator domains are widely distributed [27]. Compared with bacteria, fewer TCSs are annotated in the sequenced archaeal genomes, and TCS HKs and RRs are not always present proportionally. Within Archaea, the most TCSs are found in *Methanobacterium thermoautotrophicum* (16 HKs and 10 RRs) and *Archaeoglobus fulgidus* (14 HKs and 11 RRs) [7]. In contrast, *Pyrococcus horikoshii* contains a single HK and two RRs (chemotaxis proteins CheA, CheY, and CheB), and none are found in *Methanococcus jannaschii*, *Aeropyrum pernix*, and *Thermoplasma acidophilum* [7].

Hitherto, archaeal TCSs have not been well studied although this group of organisms is assumed to be highly adapted to extreme environments. In this work, an archaeal FilI/FilRs TCS, which is also the only *in silico* predicted TCS in the methanogenic archaeon *M. harundinacea* 6Ac, was identified experimentally, and the genes regulated directly by this TCS were determined.

TCSs are predicted to be of bacterial origin, and they were acquired by Archaea and some eukaryotes like plants, fungi, and protozoa by horizontal gene transfer [7], [15]. Though distinct from Bacteria, *Methanosaeta* 6Ac employs a TCS with properties that are similar to the bacterial system, i.e., catalyzing the phosphotransfer between FilI and FilRs, the HK and RRs of the TCS. Through ChIP-based *in vivo* assays and an EMSA-dependent *in vitro* approach, FilR1 was shown to bind the promoters of *filI* and its own gene, indicating that auto-regulation by the direct effector RR occurs in this archaeal TCS, a behavior that is typical of bacterial TCSs. FilR1 was also determined to bind to the promoters of genes involved in methanogenesis: the *acs1* operon and *acs4* gene encode acetyl-CoA synthetase for acetate activation at the initial and rate-limiting steps of acetoclastic methanogenesis; the *mtr* operon encodes a protein complex for methyl transfer from methyltetrahydromethanopterin to coenzyme M; and the *fwdCAB* operon encodes tungsten formylmethanofuran dehydrogenase subunits CABD in the methyl oxidative shunt. In addition, FilR1 was also determined to bind to the promoter of the *omp* gene, a cell envelope protein gene. These results indicate the direct regulation of acetoclastic methanogenesis and the methyl oxidative shunt by FilR1, which is inconsistent with the genes that are differentially expressed in QS-mediated filaments and short rod cells. Remarkably, a second RR protein, FilR2, is included in this archaeal TCS. This RR only has a REC domain and no output domain. The *filR2* gene constitutes an operon with the gene encoding FilI, and the two genes are cotranscribed; therefore, an equal level of the two proteins might be maintained in cells. In addition, phosphoryl group transfer occurred from FilI not only to FilR1 but also to FilR2, implying the involvement of FilR2 in this phosphorelay. However, details of its function remain to be understood. Therefore, this work demonstrates that FilI, FilR1, and FilR2 constitute a methanogen TCS that is involved in the regulation of methanogenesis.

QS regulation has been extensively studied in Bacteria [28]–[31]. Recently, a new signal transduction pathway in *Vibrio harveyi* was identified in which the QS signals, AHLs, are transduced via a TCS circuit [8], [31]–[33]. However, QS regulation seems to be overlooked in Archaea. In our previous work, FilI, presumably the N-terminal fragment including the CHASE4 domain, was shown to catalyze the synthesis of carboxyl-AHLs, the QS signal of *M. harundinacea* that regulates the cellular transition from short cells to filaments. Physiologically, filamentous cells channel the substrate acetate more to methanogenesis than to biomass, implying a decrease in the methane yield [17]. Attempting to find the link between QS and the TCS in *M. harundinacea*, differential transcription in filaments and short cells was analyzed by RNA sequencing in a separate study. The analysis indicated that genes involved in acetoclastic methanogenesis (especially *acs4*), electron transfer reactions to generate reducing equivalents, ATP synthesis, and membrane-bound transporters were all upregulated in the filaments; these results were consistent with the phenotypes (unpublished data). The experimental data in this work suggests that FilR1 directly regulates acetoclastic methanogenesis through its interactions with the promoters of the key methanogenesis genes, supporting the relationship between the FilI/FilRs TCS and QS regulation in *M. harundinacea*. Although no evidence was found for direct effects of the FilI-synthesized carboxyl-AHLs in this work, including the effect of carboxyl-AHLs on the autophosphorylation of FilI and the phosphotransfer reaction between FilI and FilRs (Figure S1), a preliminary ChIP-PCR experiment showed that FilR2 association with the promoters of *filR1*, *asc4*, *mtr*, and *omp* occurred only in the filaments. This suggests that the stand-alone RR FilR2 can act as a candidate QS-related RR in this archaeal TCS, presumably through cooperation with FilR1 or other regulators.

This work determined that the *Methanosaeta mtr*, *fwd*, *cdhCD*, and *acs4* promoters induced the transcription of a bacterial *lux* gene in *E. coli*. Because the expression was activated by the coexpression of FilR1, it mimics the natural expression in *Methanosaeta* and is unlikely to have resulted from fortuitous expression of cloned DNA near to but not including the promoter. Thus, some archaeal promoters appear to be recognized by bacterial transcription machinery, which is partially verified by mutation of the predicted promoters. This can also be anticipated on the basis of conserved promoter modularity and general basal transcription machinery among various lineages, as reviewed by Decker *et al*. [34]. Recent biochemical and structural studies have revealed significant similarities of the basal transcription machinery among Bacteria, Archaea and Eukarya that include the following: 1) structural and functional similarity of the multi-subunit polymerases; 2) conserved promoter modularity from bacteria to humans; 3) functional equivalence between bacterial σ factors and the basal transcription factors, TFB and TBP, in Archaea and Eukarya; and 4) similar strategies for transcription factors (TFs, activators, or repressors) to alter the core promoter specificity within Bacteria, Archaea, and Eukarya. While the detailed mechanisms for the action of the methanogen FilR1 with the *E. coli* transcription system need to be clarified through further studies, this work provides experimental evidence for the similarities in transcriptional machinery and regulation mechanisms between Bacteria and Archaea.

In conclusion, an archaeal FilI/FilRs TCS present in a methanogenic archaeon, *M. harundinacea*, was identified, and its regulation of methanogenesis was determined in this work. Moreover, this work indicated a possible connection between the FilI/FilRs TCS and QS regulation in this methanogenic archaeon; however, a detailed circuit pathway and mechanisms remain topics for further studies.

## Materials and Methods

### Strains, plasmids, and primers

*M. harundinacea* 6Ac was preserved in our laboratory and routinely cultured in a pre-reduced basal medium containing 100 mM sodium acetate in anaerobic bottles sealed with butyl rubber stoppers as described previously [16], [17]. *E. coli* DH5α served as the host for cloning experiments, while *E. coli* BL21 (DE3) was used for overproduction of recombinant proteins. *E. coli* strains were cultured at 37°C in Luria-Bertani (LB) medium with shaking. Kanamycin (50 µg/mL) and chloramphenicol (34 µg/mL) were supplied when necessary.

The expression vector pET28a was obtained from Novagen (Madison, WI, USA). Plasmids pCS26-pac and pACYC184 for the bioluminescence assay were kindly provided by Professor Keqian Yang (Institute of Microbiology, CAS, Beijing, China). The primers used for DNA amplification in this study (Tables S4, S5, S6) were synthesized by Sangon (Beijing, China).

### Construction of expression plasmids

The ORFs of *filR1* and *filR2* were amplified from genomic DNA of *M. harundinacea* with the primer pairs P1/P2 and P3/P4 (Table S4), respectively. The PCR products were digested with NcoI/EcoRI and NcoI/HindIII, and then they were cloned into the same sites of the vector pET28a, resulting in the expression plasmids p28FilR1 and p28FilR2, respectively. The p28FilI plasmid was used to express FilI as previously described [17]. The DNA fragment encoding the C-terminal HK domain of FilI (amino acids 642–886) was amplified from genomic DNA with the primer pair P5/P6 (Table S4) and inserted into the NheI/HindIII sites of pET28a, resulting in the expression plasmid p28FilI-C. Two ORFs (Mhar_0169 and Mhar_1520) encoding proteins with predicted REC domains served as the negative control for the phosphorelay assay and were amplified with the primer pairs P7/P8 and P9/P10 (Table S4) and cloned into plasmid pET28a to construct expression plasmids p28–0169 and p28–1520, respectively. PCR-amplified sequences were verified by DNA sequencing of all constructs.

### Protein expression and purification

To overexpress and purify FilI, FilR1, FilR2, FilI-C, Mhar_0169, and Mhar_1520, *E. coli* BL21 (DE3) harboring each of the expression plasmids was cultivated in LB medium containing kanamycin to an optical density at 600 nm of 0.4 to 0.6 when isopropyl-D-thiogalactopyranoside was added at a final concentration of 0.1–0.5 mM. The induced cultures were allowed to grow for an additional 3 h. Cells were harvested by centrifugation, resuspended in lysis buffer containing 0.3 M NaCl, 20 mM imidazole, and 50 mM sodium phosphate buffer (NaH_2_PO_4_/Na_2_HPO_4_, pH 8.0), and lysed by ultrasonication. The lysates were centrifuged at 13,400 *g* for 30 min, and the His_6_-tagged recombinant proteins were purified from the supernatants with a Ni^2+^-nitrilotriacetic acid-agarose column (Novagen), followed by ion change chromatography through a Resource Q column (GE Healthcare, Pittsburgh, PA, USA) or by gel filtration chromatography through a Superdex 200 10/300 GL column (GE Healthcare) when further purification was needed. Proteins were concentrated with Amicon Ultrafree-15 concentrators (Millipore, Billerica, MA, USA) when necessary. Purified proteins were examined by sodium dodecyl sulfate (SDS)-PAGE, and protein concentrations were determined using a bicinchoninic acid (BCA) protein concentration assay kit (Pierce, Rockford, IL, USA) combined with a Bradford protein concentration assay kit (Pierce).

### Phosphorylation assay

To test for autophosphorylation, 2 µg of recombinant FilI was incubated with 10 µCi [γ-^32^P] ATP (3000 Ci/mmol) in 10 µL of phosphorylation buffer (20 mM Tris-HCl, pH 7.5, 100 mM NaCl, 1 mM DTT, 50 mM KCl, 10 mM MgCl_2_, and 10% glycerol (v/v)) at 37°C for 45 min. For the phosphotransfer assay, 10 µL of phosphorylation buffer containing 4 µg of the recombinant FilR1 or FilR2 was subsequently added. After 2 min or 5 min, reactions were stopped by addition of 5× SDS sample buffer (250 mM Tris-HCl, pH 6.8, 5% SDS, 50% glycerol, 5% β-mercaptoethanol, 0.1% bromophenol blue) supplemented with 2.5 µL 0.5 M EDTA (pH 8.0). In control reactions, 4 µg of the recombinant Mhar_0169 and Mhar_1520 was used and incubated for 30 min or longer. The phosphorylated products were resolved by 15% SDS-PAGE, and isotope-labeled proteins were visualized by autoradiography with X-ray film.

### ChIP assay

*M. harundinacea* 6Ac was grown to the late exponential phase, corresponding to the high cell density mode of QS [17], and the cells were immediately fixed with 1% (v/v) formaldehyde for 10 min. Fixation was terminated by the addition of glycine to a final concentration of 125 mM. Culture (450 mL; ∼5×10^10^ cells) was harvested and pelleted at 10,000 *g*. Cell pellets were washed twice with Na_2_CO_3_/NaHCO_3_ buffer (50 mM Na_2_CO_3_/NaHCO_3_, 150 mM NaCl, pH 9.0), resuspended in an equal volume of Na_2_CO_3_/NaHCO_3_ buffer containing 100 mM DTT, and incubated for 2 h in anaerobic bottles. Cells were then collected at 5000 *g* and stored frozen at −80°C. Upon thawing, cells were resuspended in lysis buffer (10 mM Tris-HCl, pH 7.5, 1% (v/v) Triton X-100, 0.05% (w/v) SDS, 1 mM phenylmethylsulfonyl fluoride (PMSF)) and incubated in an ice-bath for 30 min. The protein concentration in the supernatant was determined by the BCA protein concentration assay kit (Pierce). The protein in the supernatant was diluted to 3 mg/mL with immunoprecipitation (IP) buffer (10 mM Tris-HCl, pH 7.5, 1% (v/v) Triton X-100, 0.05% (w/v) SDS, 0.05% sodium deoxycholate, 200 mM NaCl, 1 mM PMSF), and sonicated using a Bioruptor UCD300 (Diagenode, Denville, NJ, USA) until the chromatin DNA was sheared to an average size of 200–500 bp (2–7.5 min/cycle, 30 s on/30 s off, high-power setting) according to the manufacturer's instructions. Cell debris was removed by centrifugation, and the supernatant was retained.

A 500-µL aliquot of the sample was used for each IP experiment, and at least 10 µL of each sample was reserved as an input control. Each 500-µL sample was first incubated with 20 µL Dynabeads Protein A/G beads (Life Technology, Carlsbad, CA, USA) for 1 h at 4°C and mixed on a gently rotating wheel. The beads were separated from the supernatant by binding DynaMag-2 (Life Technology) and discarded. Next, the supernatant was incubated with 50 µL anti-FilR1 rabbit polyclonal antibody, which was purified by antigen affinity chromatography, overnight at 4°C with gentle mixing. A parallel experiment without antibody was conducted as a negative control (mock-IP). The supernatant was then incubated with an additional 20 µL Dynabeads Protein A/G beads that were pre-incubated with 5 mg/mL bovine serum albumin for another 1 h at 4°C with gentle mixing. Then, immunoprecipitated complex-bound beads were separated as above and washed twice with IP buffer, twice with IP buffer containing 500 mM NaCl, and once with Tris-EDTA buffer (pH 7.5).

Immunoprecipitated complexes were eluted in 53 µL Stock Reverse Crosslinking Buffer (Life Technology), combined with 1 µL proteinase K and incubated at 55°C for 60 min to de-crosslink. Control samples (10 µL) were treated with the same procedure. Samples were separated by DynaMag-2, and the beads were discarded. Supernatants and input control were then incubated at 65°C for 30 min to inactivate the proteinase K. Finally, the non-crosslinked DNA was purified using DNA Purification Magnetic Beads and the provided buffers (Life Technology) or by the QIAquick PCR Purification Kit (Qiagen, Dusseldorf, Germany). DNA concentrations were determined by the Quant-iT DNA Assay Kit (Life Technology), and DNA fragment sizes were checked by an Agilent 2100 Bioanalyzer. All ChIP assays were performed at least three times to ensure the reproducibility.

### PCR and real-time qPCR assay

PCR and real-time qPCR were performed to determine the enrichment of FilR1-bound targets in the immunoprecipitated DNA samples. For PCR amplification, 1 µL input, IP, or mock-IP (CK) DNA samples were used in a 25-µL reaction mix containing 0.4 µM of each oligonucleotide primer. PCR amplification used rTaq DNA polymerase (Takara, Dalian, China) for 25 to 30 cycles. Then, 5–10 µL reaction mixture was analyzed by electrophoresis on a 1.5% agarose gel. For qPCR analysis, a 25-µL reaction mixture was prepared that contained 1×SYBR Green Real-time PCR Master Mix (Toyobo, Tokyo, Japan), 0.4 µM each of forward and reverse primers (Table S4), and 1 µL input, IP, or mock-IP (CK) DNA samples as templates. The qPCR analysis was performed on a Mastercycler ep realplex real-time PCR machine (Eppendorf, Hamburg, Germany). Fold differences between samples were calculated as described previously [35]. Briefly, the ΔCt value (normalized to the input samples) for each sample was calculated according to the equation: ΔCt [Ct (sample) − Ct (input)]. Next, the ΔΔCt was calculated by ΔΔCt = ΔCt (IP sample) − ΔCt (mock-IP control). Finally, the fold difference between the IP sample and mock-IP control was calculated as 2^(−ΔΔCt)^.

### EMSA

All the probes that were used were amplified with a biotin-labeled forward primer. Double-stranded DNA (dsDNA) probes targeting the intergenic regions upstream of the studied genes (Table S1) including their DNA promoter regions were amplified using the genomic DNA of *M. harundinacea*as as the template (Table S5), while the ORF of *filI* and the predicted DNA promoter region of Mhar_0449 were amplified as control probes. The DNA probes generated via PCR amplification were purified by an agarose gel DNA Purification Kit (Qiagen). A standard EMSA was performed using a Light Shift Chemiluminescent EMSA Kit (Pierce) as recommended by the manufacturer with some modifications as follows. A standard binding reaction mixture (20 µL) contained 10 mM Tris-HCl (pH 8.0), 50 mM KCl, 10 mM MgCl_2_, 1 mM DTT, 2.5% glycerol, 1% Nonidet P 40, 0.5 mM EDTA, 1 µg/µL poly dI-dC, 10 fmol labeled DNA probe, and the indicated amount of purified His_6_-FilR1 used in each reaction. After incubating at 25°C for 20 min, samples were immediately loaded on a non-denaturing 5% polyacrylamide gel (with an acrylamide to bisacrylamide weight ratio of 80∶1) in 0.5× Tris-borate-EDTA buffer and run at 150 V for 2.5 h. Then, the DNA-protein complex was transferred onto a nylon membrane and cross-linked using a GS Gene Linker UV Chamber (Bio-Rad, Hercules, CA, USA). The biotin-labeled DNA was detected by chemiluminescence.

To determine the effect of phosphorylation on FilR DNA-binding, FilI protein (5 pmol) was autophosphorylated by incubation with 50 pmol ATP for 45 min at 37°C. Then, autophosphorylated FilI was mixed with 0, 0.1, 0.5, 1, and 2.5 pmol FilR1 for 10 min. EMSA of the phosphorylated FilR1 protein was performed as above.

### Construction of promoter-reporter strains for luciferase assays in *E. coli*

*E. coli*-based promoter-reporter strains were constructed to detect the possible regulation of genes of interest by FilR1. Intergenic regions upstream of the tested genes including their promoters were amplified by PCR with primer pairs listed in Table S6 and genomic DNA of *M. harundinacea* as a template. The amplified DNA fragments were then ligated to BamHI/XhoI-digested pCS26-Pac to yield the reporter plasmids pO^x^-lux (x refers to the tested gene). To express the FilR1 protein in *E. coli*, the *filR1* gene was amplified with the primer pair P37/P38 (Table S6) using the plasmid p28FilR as a template and introducing *E. coli* Shine-Dalgarno sequences and recognition sites for BamHI [23]. After digestion with BamHI, the fragment was ligated to BamHI/EcoRV-digested pACYC184 to yield the plasmid pFilR1. AT-tract mutants were constructed by PCR with primer pairs listed in Table S6 and pO^x^-lux reporter plasmids as templates; PCR products were digested with DpnI to yield the pO^x-mut^-lux reporter plasmids. The bioluminescence of the *E. coli* reporter cultures was measured using a TD 20/20n single tube luminometer (Turner Biosystems, Sunnyvale, CA, USA) as described previously [23]. All measurements were performed with duplicate samples, and all experiments were repeated at least three times.

## Supporting Information

Figure S1 **Effect of FilI synthetic carboxyl-AHLs on the autophosphorylation of FilI and phosphotransfer from FilI to FilRs visualized on SDS-PAGE.** (A) autophosphorylation of the recombinant FilI protein (2 µg) incubated with [γ-^32^P]ATP for 45 min at 37°C in the presence (+) or absence (−) of a carboxyl-AHL (*N*-carboxyl-C_10_-HSL,m/z 318,or *N*-carboxyl-C_12_-HSL, m/z 346 at final concentration 2 ng). (B) Phosphotransfer of the autophosphorylated FilI to His-tagged RRs (4 µg) for 5 min in the presence (+) or absence (−) of carboxyl-AHLs (*N*-carboxyl-C10-HSL,m/z 318,or *N*-carboxyl-C12-HSL, m/z 346 at final concentration 2 ng). Autophosphorylation and phosphotransfer reactions without FilI included as negative controls. Solid arrows indicate the phosphorylated proteins: pi-FilI, pi-FilR1 and pi-FilR2. Dotted arrows indicate nonspecific bands.
(TIF)

Figure S2 **EMSAs showed phosphorylation enhancing the DNA binding affinity of FilR1.** FilI protein (5 pmol) was firstly autophosphorylated by incubation with ATP (50 pmol) for 45 min at 37°C, and then mixed with 0, 0.1, 0.5, 1, 2.5 pmol FilR1 for 10 min, respectively. The mixtures including phosphorylated FilR1 protein were incubated with 0.5 nM of biotin-labeled DNA in the standard binding reaction mixture at 25°Cfor 20 min before electrophoresis on native PAGE. The final concentrations of the FilR1 proteins for EMSA were shown at the top of each lane. (A) P*_acs1_*, promoter of the *acs1* operon; (B) P*_fwd_*, promoter of the *fwd* operon. FilR1, purified recombination FilR1 protein; Pi-FilR1, FilR1 protein incubated with autophosphorylated FilI protein before EMSA.
(TIF)

Figure S3 **Schematic architecture of the predicted promoter of** ***mtr*** **.** Two transcription start sites (TSS), TSS1 (at −20 nt) and TSS2 (defined as +1), were predicted upstream the *mtr* coding region. Predicted TATA box, BRE and bacteria promoter character −35 region are shadowed, and the distances (nt) from TSS2 are indicated. TATA box (AATTAA) was mutated by a substitution of GGACCC in the experiment of *E. coli* RNA polymerase recognizing archaeal promoters.
(TIF)

Table S1 **Genes of** ***M. harundinacea*** **6Ac studied.**
(PDF)

Table S2 **Promoter searching for some genes of** ***M. harundinacea*** **6Ac using Neural Network Promoter Prediction Program.**
(PDF)

Table S3 **Effect of mutation of predicted TATA box in archaeal promoters on their expression in the** ***E. coli ex vivo*** **reporter system.**
(PDF)

Table S4 **Primers used for construction of expression plasmids.**
(PDF)

Table S5 **Primers used for amplification of promoter regions of the tested genes.**
(PDF)

Table S6 **Primer pairs used for construction of reporter plasmids for luciferase assays.**
(PDF)
