# Supplementary figures and images for: Characterization of an Archaeal Two-Component System That Regulates Methanogenesis in Methanosaeta harundinacea
Source: PLoS One. 2014 Apr 18;9(4):e95502. doi: 10.1371/journal.pone.0095502 (PMC3991700; doi:10.1371/journal.pone.0095502)

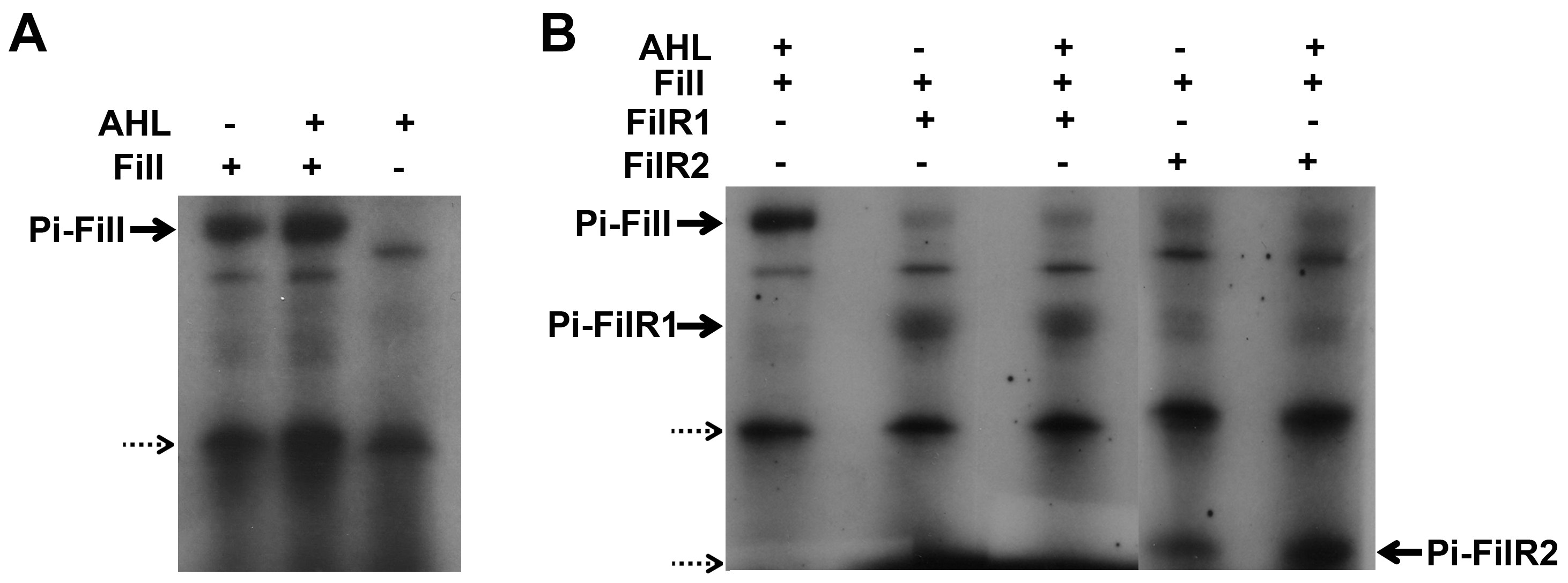

Supplement: Figure S1 — Effect of FilI synthetic carboxyl-AHLs on the autophosphorylation of FilI and phosphotransfer from FilI to FilRs visualized on SDS-PAGE. (A) autophosphorylation of the recombinant FilI protein (2 µg) incubated with [γ-32P]ATP for 45 min at 37°C in the presence (+) or absence (−) of a carboxyl-AHL (N-carboxyl-C10-HSL,m/z 318,or N-carboxyl-C12-HSL, m/z 346 at final concentration 2 ng). (B) Phosphotransfer of the autophosphorylated FilI to His-tagged RRs (4 µg) for 5 min in the presence (+) or absence (−) of carboxyl-AHLs (N-carboxyl-C10-HSL,m/z 318,or N-carboxyl-C12-HSL, m/z 346 at final concentration 2 ng). Autophosphorylation and phosphotransfer reactions without FilI included as negative controls. Solid arrows indicate the phosphorylated proteins: pi-FilI, pi-FilR1 and pi-FilR2. Dotted arrows indicate nonspecific bands. (TIF) [file pone.0095502.s001.tif]

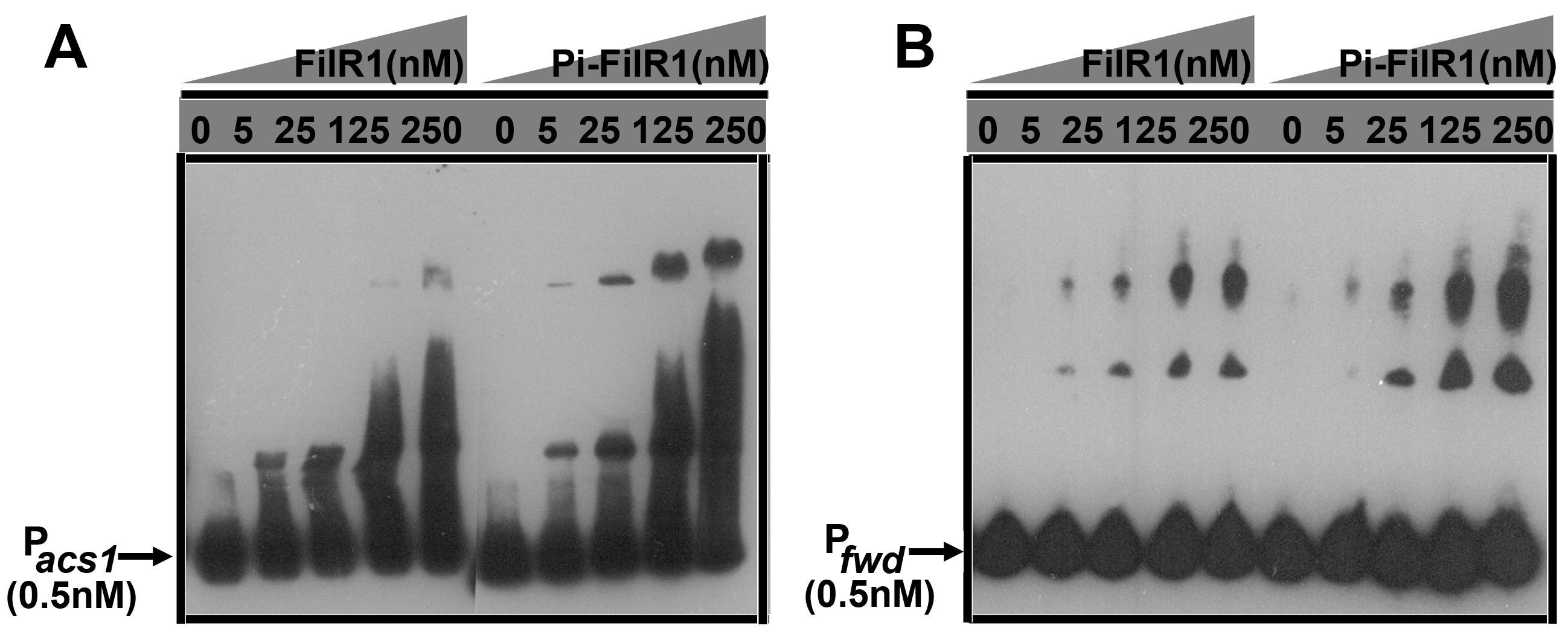

Supplement: Figure S2 — EMSAs showed phosphorylation enhancing the DNA binding affinity of FilR1. FilI protein (5 pmol) was firstly autophosphorylated by incubation with ATP (50 pmol) for 45 min at 37°C, and then mixed with 0, 0.1, 0.5, 1, 2.5 pmol FilR1 for 10 min, respectively. The mixtures including phosphorylated FilR1 protein were incubated with 0.5 nM of biotin-labeled DNA in the standard binding reaction mixture at 25°Cfor 20 min before electrophoresis on native PAGE. The final concentrations of the FilR1 proteins for EMSA were shown at the top of each lane. (A) Pacs1, promoter of the acs1 operon; (B) Pfwd, promoter of the fwd operon. FilR1, purified recombination FilR1 protein; Pi-FilR1, FilR1 protein incubated with autophosphorylated FilI protein before EMSA. (TIF) [file pone.0095502.s002.tif]

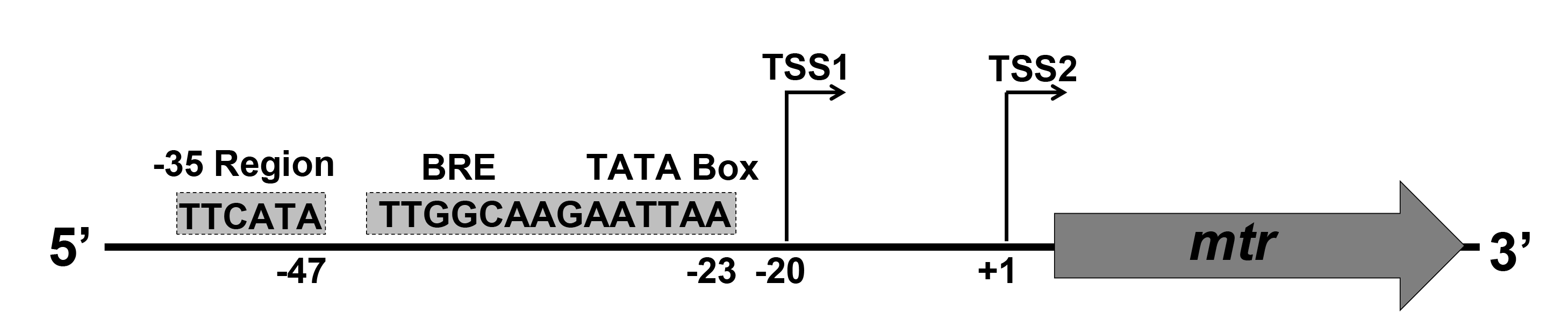

Supplement: Figure S3 — Schematic architecture of the predicted promoter of mtr . Two transcription start sites (TSS), TSS1 (at −20 nt) and TSS2 (defined as +1), were predicted upstream the mtr coding region. Predicted TATA box, BRE and bacteria promoter character −35 region are shadowed, and the distances (nt) from TSS2 are indicated. TATA box (AATTAA) was mutated by a substitution of GGACCC in the experiment of E. coli RNA polymerase recognizing archaeal promoters. (TIF) [file pone.0095502.s003.tif]
