# Supplementary material for: Characterization of an Archaeal Two-Component System That Regulates Methanogenesis in Methanosaeta harundinacea
Source: PLoS One. 2014 Apr 18;9(4):e95502. doi: 10.1371/journal.pone.0095502 (PMC3991700; doi:10.1371/journal.pone.0095502)
Supplement: Table S1 — Genes of M. harundinacea 6Ac studied. (PDF) [file pone.0095502.s004.pdf]

Table S1. Genes of *M. harundinacea* 6Ac studied

Annotation (gene or operon)	Orf number	Gene product
fwdCABD	Mhar_0373	FwdC (Tungsten formylmethanofuran dehydrogenase, subunit C)
	Mhar_0374	FwdA (Tungsten formylmethanofuran dehydrogenase, subunit A)
	Mhar_0375	FwdB ((Tungsten formylmethanofuran dehydrogenase, subunit B)
	Mhar_0376	FwdD (Tungsten formylmethanofuran dehydrogenase, subunit D)
RNA pol	Mhar_0380	DNA-directed RNA polymerase, subunit H
	Mhar_0381	DNA-directed RNA polymerase, subunit B'
	Mhar_0382	DNA-directed RNA polymerase, subunit B'
	Mhar_0383	DNA-directed RNA polymerase subunit A'
	Mhar_0384	DNA-directed RNA polymerase subunit A"
	Mhar_0385	50S ribosomal protein L30e
	Mhar_0386	Transcription termination factor NusA
filR1	Mhar_0445	FilR1
filI-filR2	Mhar_0446	FilI
	Mhar_0447	FilR2
Mhar_0449	Mhar_0449	hypothetical protein
mcrBD	Mhar_0495	McrB (Methyl-coenzyme M reductase, beta subunit)
	Mhar_0496	McrD (Methyl-coenzyme M reductase, D subunit)
mcrCA	Mhar_0497	McrC (Methyl-coenzyme M reductase, gamma subunit)
	Mhar_0498	McrA (Methyl-coenzyme M reductase, alpha subunit)
acs1	Mhar_0583	Ketoisovalerateferredoxinoxidoreductase, β subunit
	Mhar_0584	Ketoisovalerateferredoxinoxidoreductase, alpha subunit
	Mhar_0585	Acs1 (AMP-forming acetyl-CoA synthetase)
	Mhar_0586	Transcriptional regulator
acs4	Mhar_0752	Acs4 (Acetyl-coenzyme A synthetase)
fpo	Mhar_1410	FpoA (F420H2 dehydrogenase, subunit A)
	Mhar_1411	FpoB (F420H2 dehydrogenase, subunit B)
	Mhar_1412	FpoD (F420H2 dehydrogenase, subunit D)
	Mhar_1413	FpoH (F420H2 dehydrogenase, subunit H)
	Mhar_1414	FpoI (F420H2 dehydrogenase, subunit I)
	Mhar_1415	FpoJ (F420H2 dehydrogenase, subunit J)
	Mhar_1416	FpoJ (F420H2 dehydrogenase, subunit J)
	Mhar_1417	FpoK (F420H2 dehydrogenase, subunit K)
	Mhar_1418	FpoL (F420H2 dehydrogenase, subunit L)
	Mhar_1419	FpoM (F420H2 dehydrogenase, subunit M)
	Mhar_1420	FpoN (F420H2 dehydrogenase, subunit N)
mtr	Mhar_2090	MtrE (Tetrahydromethanopterin S-methyltransferase, subunit E)
	Mhar_2091	MtrD (Tetrahydromethanopterin S-methyltransferase, subunit D)
	Mhar_2092	MtrC (Tetrahydromethanopterin S-methyltransferase, subunit C)
	Mhar_2093	MtcB (Tetrahydromethanopterin S-methyltransferase, subunit B)
	Mhar_2094	MtrA (Tetrahydromethanopterin S-methyltransferase, subunit A)
	Mhar_2095	MtrF (Tetrahydromethanopterin S-methyltransferase subunit F)

	Mhar_2096	MtrG (Tetrahydromethanopterin S-methyltransferase, subunit G)
	Mhar_2097	MtrH (Tetrahydromethanopterin S-methyltransferase, subunit H)
omp	Mhar_2112	Outer membrane protein
	Mhar_2322	CdhC (Acetyl-CoA decarbonylase/synthase gamma subunit)
cdhCD	Mhar_2323	CdhD (Acetyl-CoA decarbonylase/synthase delta subunit)
	Mhar_2324	CO dehydrogenase maturation factor
	Mhar_2325	CdhC (CO dehydrogenase/acetyl-CoA synthase C subunit)
	Mhar_2326	CdhB (Acetyl-CoA decarbonylase/synthase beta subunit)
cdhBEA	Mhar_2327	CdhE(Acetyl-CoA decarbonylase/synthase epsilon subunit)
	Mhar_2328	CdhA (Acetyl-CoA decarbonylase/synthase alpha subunit)
