# Supplementary material for: Characterization of an Archaeal Two-Component System That Regulates Methanogenesis in Methanosaeta harundinacea
Source: PLoS One. 2014 Apr 18;9(4):e95502. doi: 10.1371/journal.pone.0095502 (PMC3991700; doi:10.1371/journal.pone.0095502)
Supplement: Table S2 — Promoter searching for some genes of M. harundinacea 6Ac using Neural Network Promoter Prediction Program. (PDF) [file pone.0095502.s005.pdf]

Table S2. Promoter searching for some genes of *M. harundinacea* 6Ac using Neural Network Promoter Prediction Program.

Gene	Predicted Promoter sequence[a]	Score
filR1	atccttcctcttatctggggctctatcgccacgacggagtcTacgacgtgg	0.58
filI-filR2	agggttatcttcataaaccacggacggatgacagtaaggcAggtgcgagg	0.69
acs1	ggggggtttattaataggttgaggtccccctctcgaggccCggaaagggc	0.73
acs4	attttcaggatttatacccggccgatattccatattttggAatctataat	0.9
mtr	attactatgatttggcaagaattaaaaaggaggagagcaaatggacaTggttagc	0.99
omp	atggatgatccttcaaagggcggtccacctgacgatccccCtcatagctg	0.69
fwd	tccccagggctaaatattgtgcggtcgtaccctggggacgGtcactttaa	0.98

a. Capital letters refer to the predicted transcriptional start site (TSS), except that the TSS of *mtr* is identified by a transcriptomic study; predicted TATA boxes are underlined.
