# Supplementary material for: Characterization of an Archaeal Two-Component System That Regulates Methanogenesis in Methanosaeta harundinacea
Source: PLoS One. 2014 Apr 18;9(4):e95502. doi: 10.1371/journal.pone.0095502 (PMC3991700; doi:10.1371/journal.pone.0095502)
Supplement: Table S3 — Effect of mutation of predicted TATA box in archaeal promoters on their expression in the E. coli ex vivo reporter system. (PDF) [file pone.0095502.s006.pdf]

Table S3. Effect of mutation of predicted TATA box in archaeal promoters on their expression in the *E. coli ex vivo* reporter system

Reporter plasmids[a]	Bioluminescence[b] by + p184[c]		Bioluminescence by + pFilR1[d]	
	Wild type promoter	AT-rich mutant	Wild type promoter	AT-rich mutant
pO*filR1* -lux	437±68	529±84	3334±517	3283±536
pO*filI-filR2*-lux	1985±466	310±98	66330±6361	713±79
pO*Acs4*-lux	169013±4054	196060±18531[e]	7758086±61087	7559091±103678[e]
		184595±15634[f]		7612925±305210[f]
pO*Mtr* -lux	879377±60268	27907±5215	3247045±67755	255856±34811

a. Annotations of each reporter plasmid were the same as listed in Table S4.

b. Values are shown as relative light units of the average from at least three independent readings.

c. *E. coli* strain carrying plasmid pairs of FilR1-vacant p184 plus pO^{x}-lux.

d. *E. coli* strain carrying plasmid pairs of FilR1- p184 plus pO^{x}-lux.

e. Mutant with substitution at the upstream AT-rich region listed in Table S2.

f. Mutant with substitution at the downstream AT-rich region listed in Table S2.
