# Supplementary material for: Characterization of an Archaeal Two-Component System That Regulates Methanogenesis in Methanosaeta harundinacea
Source: PLoS One. 2014 Apr 18;9(4):e95502. doi: 10.1371/journal.pone.0095502 (PMC3991700; doi:10.1371/journal.pone.0095502)
Supplement: Table S4 — Primers used for construction of expression plasmids. (PDF) [file pone.0095502.s007.pdf]

Table S4. Primers used for construction of expression plasmids

Names	Sequence (5’ to 3’) a	Application
P1	actccatggacgaaagggatatggt	Construction of p28FilR1
P2	acggaattcaccttgagggggagg	
P3	actccatggtgagctacggcctgga	Construction of p28FilR2
P4	gacaagcttcgcgggtccggggg	
P5	ctagctagccggacgagggcggacc	Construction of p28FilI-C
P6	cccaagctttcagggggtcgggagg	
P7	catgccatgggggatgatcctggaa	Construction of p28-0169
P8	cccaagcttatatctatcctggggtgggc	
P9	catgccatggctgcggcgattc	Construction of p28-1520
P10	cccaagctttctggagacgtagctgag	

a. Restriction sites are underlined.
