# Supplementary material for: Characterization of an Archaeal Two-Component System That Regulates Methanogenesis in Methanosaeta harundinacea
Source: PLoS One. 2014 Apr 18;9(4):e95502. doi: 10.1371/journal.pone.0095502 (PMC3991700; doi:10.1371/journal.pone.0095502)
Supplement: Table S5 — Primers used for amplification of promoter regions of the tested genes. (PDF) [file pone.0095502.s008.pdf]

**Table S5. Primers used for amplification of promoter regions of the tested genes**

| Names | Sequence (5' to 3')[a] | Application |
|---|---|---|
| P49 | cggtacgatgagaacttc | Amplifying promoter region of *filR1* |
| P50 | acccgtttgaccatatcc | |
| P51 | agatgaaaccagaattat | Amplifying promoter region of *filI-filR2* |
| P52 | cttcctttcaacctcctc | |
| P53 | aagccctgcctgccaaag | Amplifying promoter region of *fwdCABD* |
| P54 | tcaccatcagattcaaac | |
| P55 | aggttttatgccggatga | Amplifying promoter region of RNA pol |
| P56 | tcttcatacagccacttc | |
| P57 | gccacatagtccaggtct | Amplifying promoter region of *mcrBD* |
| P58 | cacgttctttttcctcct | |
| P59 | ggccattttgtctgatac | Amplifying promoter region of *mcrCA* |
| P60 | gcttctccagatcgctag | |
| P61 | aacgggttccatctcgat | Amplifying promoter region of *acs1* |
| P62 | ctccgccatgccgattca | |
| P63 | ccattaggagccgtcgat | Amplifying promoter region of *acs4* |
| P64 | agccaatttcaaccctcc | |
| P65 | aatccacctaaactcacc | Amplifying promoter region of *fpo* |
| P66 | caccaattcgccgctcat | |
| P67 | ttcgccccaaactgttct | Amplifying promoter region of *mtr* |
| P68 | gctaaccatgtccatttg | |
| P69 | gggcaaaacctctcctcg | Amplifying promoter region of *omp* |
| P70 | aacccccataaatgatag | |
| P71 | gaatttatagtatctctg | Amplifying promoter region of *cdhCD* |
| P72 | ccatccctctccctcaag | |
| P73 | aaaccgccctcctctcca | Amplifying promoter region of *cdhBEA* |
| P74 | gcttgacctccttctgta | |
| P75 | ggtcggagaggctgaggg | Amplifying promoter region of *filI* |
| P76 | ggcgcagcgtcatgggtg | |
| P77 | gtgccgaggtagtctagt | Amplifying promoter region of Mhar_0449 |
| P78 | attccacctcctcatatc | |

a. The first primer of each primer pair was labelled by biotin at the 5' terminal.
