# Supplementary material for: Characterization of an Archaeal Two-Component System That Regulates Methanogenesis in Methanosaeta harundinacea
Source: PLoS One. 2014 Apr 18;9(4):e95502. doi: 10.1371/journal.pone.0095502 (PMC3991700; doi:10.1371/journal.pone.0095502)
Supplement: Table S6 — Primer pairs used for construction of reporter plasmids for luciferase assays. (PDF) [file pone.0095502.s009.pdf]

Table S6. Primer pairs used for construction of reporter plasmids for luciferase assays.

Names	Sequence (5' to 3') [a]	Application
P11	ccactcgagcggtacgatgagaacttc	Construct pOfilR1-lux
P12	agaggatccacccgtttgaccatatcc	
P13	ccactcgagagatgaaaccagaattat	Construct pOfilI-filR2-lux
P14	agaggatcccttcctttcaacctcctc	
P15	ccactcgagaagccctgcctgccaaag	Construct pOFwdCABD-lux
P16	agaggatcctcaccatcagattcaaac	
P17	ccactcgagaggttttatgccggatga	Construct pORNA pol-lux
P18	agaggatcctcttcatacagccacttc	
P19	ccactcgaggccacatagtccaggtct	Construct pOMcrBD-lux
P20	agaggatcccacgttctttttcctcct	
P21	ccactcgagggccattttgtctgatac	Construct pOMcrCA-lux
P22	agaggatccgcttctccagatcgctag	
P23	ccactcgagaacgggttccatctcgat	Construct pOAcs1-lux
P24	agaggatccctccgccatgccgattca	
P25	ccactcgagccattaggagccgtcgat	Construct pOAcs4lux
P26	agaggatccagccaatttcaaccctcc	
P27	ccactcgagaatccacctaaactcacc	Construct pOFpo-lux
P28	agaggatcccaccaattcgccgctcat	
P29	ccactcgagttcgccccaaactgttct	Construct pOMtr-lux
P30	agaggatccgctaaccatgtccatttg	
P31	ccactcgaggggcaaaacctctcctcg	Construct pOOmp-lux
P32	agaggatccaacccccataaatgatag	
P33	ccactcgaggaatttatagtatctctg	Construct pOCdhCD-lux
P34	agaggatccccatccctctccctcaag	
P35	ccactcgagaaaccgccctcctctcca	Construct pOCdhBEA-lux
P36	agaggatccgcttgacctccttctgta	
P37	tttaagaaggagatatac	Construct pFilR1
P38	tgaggatcctcatcagaccttgagg	
P39	tcactgaatccttccGACCCGcggggctctatcgcca	Construct pO$^{\mathit{filR1}\text{-mut1-lux}}$
P40	tggcgatagagccccGCGGGTCggaaggattcagtca	
P41	actcggagggttatcGGGACCCGcacggacggatgaca	Construct pO$^{\mathit{filI\text{-}filR2}\text{-mut1-lux}}$
P42	tgtcatccgtccgtggCGGGTCCCgataaccctccgagt	
P43	ttttgattttcaggaGACCCGcccggccgatattcca	Construct pO$^{\mathit{acs4}\text{-mut1-lux}}$
P44	tggaatatcggccgggCGGGTCTcctgaaaatcaaaa	
P45	atttatacccggccGGACCCGcatattttggaatcta	Construct pO$^{\mathit{acs4}\text{-mut2-lux}}$
P46	tagattccaaaatatGCGGGTCcggccgggtataaat	

P47	tactatgatttggcaCGGACCCGaaaggaggagagcaa	Construct $pO^{mtr\text{-mut-lux}}$
P48	ttgctctcctctcctcctttcGGGTCCGTgccaaatcatacta	

a. Restriction sites are underlined, and the capital letters in the sequence represent the mutant nucleotides.
